# Supplementary material for: The state of the leprosy epidemic in Yunnan, China 2011–2020: A spatial and spatiotemporal analysis, highlighting areas for intervention
Source: PLoS Negl Trop Dis. 2021 Oct 6;15(10):e0009783. doi: 10.1371/journal.pntd.0009783 (PMC8494331; doi:10.1371/journal.pntd.0009783)
Supplement: Attachment — Submitted filename: Revised.doc [file pntd.0009783.s001.doc]

---Original---

**From:** "PLOS Neglected Tropical Diseases"<[em@editorialmanager.com](mailto:em@editorialmanager.com)>

**Date:** Wed, Aug 4, 2021 10:13 AM

**To:** "tiejun Shui"<[67637539@qq.com](mailto:67637539@qq.com)>;

**Subject:** Decision on your PLOS Neglected Tropical Diseases submission (PNTD-D-21-00874) - [EMID:6c1d07d7950a1439]

CC: [carlos.franco.paredes@gmail.com](mailto:carlos.franco.paredes@gmail.com), [gerson.penna@fiocruz.br](mailto:gerson.penna@fiocruz.br), [gpenna@gpenna.net](mailto:gpenna@gpenna.net)

Dear Mrs. tiejun Shui,
 
Thank you very much for submitting your manuscript "The state of the  leprosy  epidemic in  Yunnan ,  China 2011-2020: a spatial and spatial-temporal analysis and highlight areas for intervention" for consideration at PLOS Neglected Tropical Diseases. As with all papers reviewed by the journal, your manuscript was reviewed by members of the editorial board and by several independent reviewers. The reviewers appreciated the attention to an important topic. Based on the reviews, we are likely to accept this manuscript for publication, providing that you modify the manuscript according to the review recommendations.
 

Please prepare and submit your revised manuscript within 30 days. If you anticipate any delay, please let us know the expected resubmission date by replying to this email. 

When you are ready to resubmit, please upload the following:

[1] A letter containing a detailed list of your responses to all review comments, and a description of the changes you have made in the manuscript.
Please note while forming your response, if your article is accepted, you may have the opportunity to make the peer review history publicly available. The record will include editor decision letters (with reviews) and your responses to reviewer comments. If eligible, we will contact you to opt in or out

[2] Two versions of the revised manuscript: one with either highlights or tracked changes denoting where the text has been changed; the other a clean version (uploaded as the manuscript file).

Important additional instructions are given below your reviewer comments.

Thank you again for your submission to our journal. We hope that our editorial process has been constructive so far, and we welcome your feedback at any time. Please don't hesitate to contact us if you have any questions or comments.
 
Sincerely,

Carlos Franco-Paredes
Associate Editor
PLOS Neglected Tropical Diseases

Gerson Penna
Deputy Editor
PLOS Neglected Tropical Diseases

***********************


Reviewer's Responses to Questions

<b>Key Review Criteria Required for Acceptance?</b></br></br>
As you describe the new analyses required for acceptance, please consider the following:</br></br>

<b>Methods</b></br></br>
-Are the objectives of the study clearly articulated with a clear testable hypothesis stated?</br>
-Is the study design appropriate to address the stated objectives?</br>
-Is the population clearly described and appropriate for the hypothesis being tested?</br>
-Is the sample size sufficient to ensure adequate power to address the hypothesis being tested?</br>
-Were correct statistical analysis used to support conclusions?</br>
-Are there concerns about ethical or regulatory requirements being met?</br></br>

Reviewer #1: Authors need to make adjustments to the statistical method.

Reviewer #2: 1. Although the study design is appropriate to address the stated objectives, however, how did the author consider some factors such as floating population of new cases which maybe cause the different results.

Reply: Thanks for reviewer’s comments.

In Yunnan China, almost all of the leprosy cases were resident of Yunnan, seldom cases from other province of China or other country.

2. All the data from LEPMIS should be confirmed by the national and provincial experts.
Reply: Thanks for reviewer’s comments.

Yes. All of the leprosy cases were confirmed by the the provincial or national experts

Reviewer #3: Methods are appropriated to the objectives traced for the study.

--------------------

<b>Results</b></br></br>
-Does the analysis presented match the analysis plan?</br>
-Are the results clearly and completely presented?</br>
-Are the figures (Tables, Images) of sufficient quality for clarity?</br></br>

Reviewer #1: The results tables and figures need adjustments.

Reviewer #2: 1. Line 142-144"The number of newly detected leprosy cases were declined from 283 newly detected leprosy patients reported in 1990, to 119 cases reported at the end of 2020." Are the 283 newly detected leprosy patients reported in 2011?

Reply: Thanks for reviewer’s comments.

It has been revised from “1990” to “2011”.

2. All figures are not very clear, and should be more clear.
Reply: Thanks for reviewer’s comments.

All of the figures had been uploaded to the website <https://pacev2.apexcovantage.com/.>, and meet the requirements of journal of PLOS NTD to check the quality.

Reviewer #3: Yes for all questions
Reply: Thanks for reviewer’s comments.

--------------------

<b>Conclusions</b></br></br>
-Are the conclusions supported by the data presented?</br>
-Are the limitations of analysis clearly described?</br>
-Do the authors discuss how these data can be helpful to advance our understanding of the topic under study?</br>
-Is public health relevance addressed?</br></br>

Reviewer #1: Please, see "Summary and General Comments"

Reviewer #2: "The epidemic of leprosy in Yunnan decreased totally. While there were still part of regions with high NCDR and/or clustering regions." What's the reason? it didn't analyze very clear, especially in some areas with stable high NCDR.

Reply: Thanks for reviewer’s comments.

In this study, we used ArcGIS and other software to indicate the highlight areas for intervention. For the reason of with high NCDR and/or clustering, which may due to remain inadequate early detection, and as multibacillary (MB) was the majority type, which may indicated the transmission was still existing in the community in Yunnan, China.

Reviewer #3: Yes for all questions
Reply: Thanks for reviewer’s comments.

--------------------

<b>Editorial and Data Presentation Modifications?</b></br><br/>
Use  this section for editorial suggestions as well as relatively minor modifications of existing data that would enhance clarity. If the only modifications needed are minor and/or editorial, you may wish to recommend “Minor Revision” or “Accept”.

Reviewer #1: (No Response)

Reviewer #2: Minor Revision

Reviewer #3: (No Response)
Reply: Thanks for reviewer’s comments.
--------------------

<b>Summary and General Comments</b></br></br>
Use  this section to provide overall comments, discuss strengths/weaknesses of the study, novelty, significance, general execution and scholarship. You may also include additional comments for the author, including concerns about dual publication, research ethics, or publication ethics. If requesting major revision, please articulate the new experiments that are needed.

Reviewer #1: Abstract:

According Author Guidelines: “The Abstract is conceptually divided into the following three sections with these headings: Background, Methodology/Principal Findings, and Conclusions/Significance.”

Reply: Thanks for reviewer’s comments.
It has been revised into three parts: Background, Methodology/Principal Findings, and Conclusions/Significance.

Recommendations: Background is missing in manuscript.
Reply: Thanks for reviewer’s comments.

It was revised as: Background: Despite public health efforts to reduce the leprosy burden in Yunnan, china, leprosy remains an important public health problem in some pockets areas.

Introduction:

According authors’ manuscript:

(57-59) “Although the World Health Organization (WHO) elimination target has been achieved in 2000, 58 with a global prevalence rate of <1 case/10,000 people leprosy remains an important public health 59 problem in some low and middle-income countries”

Consideration: Not all countries have achieved the goal of "eliminating leprosy" proposed by the WHO, including Brazil, which is a large country and has an expressive contribution to maintaining the number of leprosy cases. This reviewer suggests an introduction that discusses the aspects that led to the declaration of elimination of leprosy, especially in hyperendemic countries.
Reply: Thanks for reviewer’s comments.
It was revised in introduction, line 58-62: Despite being declared "eliminated" as a global public health problem by the World Health Organization in the year 2000, with a global prevalence rate of <1 case/10,000 people, leprosy remains an important public health problem in some low and middle-income countries [3] and some specific high-burden pockets [4].[5], including India Brazil, and Indonesia, which took many more years to reach the national elimination target of <1 case per 10,000 persons, and account for most new cases (80.20 %) [ref].

[Ref] Maymone MBC, Laughter M, Venkatesh S, Dacso MM, Rao PN, Stryjewska BM, Hugh J, Dellavalle RP, Dunnick CA. Leprosy: Clinical aspects and diagnostic techniques. J Am Acad Dermatol. 2020 Jul;83(1):1-14. doi: 10.1016/j.jaad.2019.12.080. Epub 2020 Mar 27. PMID: 32229279.

Statistical Analysis:

According authors’ manuscript: (121-122) “Grade 2 deformity was defined as visible disability”

Consideration: There is a conceptual inversion in this sentence. There is a conceptual inversion in this sentence. The correct sentence would be degree 2 of disability not as visible deformity.
The reference used was (30) WHO Expert Committee on Leprosy, World Health Organization. WHO Expert Committee on leprosy [meeting held in Geneva from 17 to 24 November 1987]: Sixth report. Geneva, 465 Switzerland: World Health Organization; 1988.
However, according WHO Expert Committee on Leprosy: seventh report (1).
“Committee endorsed this grading with the amendment that lagophthalmos. iridocyclitis and corneal opacities be considered as grade 2”.
This aspect is critical, was not taken into consideration, and may have led to underestimated results concerning the evaluated population. The same mistake is found in the caption of table 1.
Reply: Thanks for reviewer’s comments.

The criteria of G2D was revised as according to reference:

World Health Organization Expert Committee on Leprosy: Seventh report. Technical Report Series 847. Geneva, 1998.

Results and discussion:

According authors’ manuscript:

(151-154): “A total of 1279 MB cases were reported in Yunnan Province during 2011–2020, which accounted for 67.07% of new detected leprosy cases in the whole province. The number of MB cases was declined year by year from 172 in 2011 to 96 in 2020, while the ratio of MB cases was increasing year by year, which ranged from 60.78% in 2011 to 80.67% in 2020”

(160-161) “The rate of G2D gradually decreased to 10.34% in 2018, while increased to 12.50% in 2019, but further decreased to 10.08% in 2020”

(167-168) “The prevalence of leprosy among children fluctuated from 0.63% to 5.35%.”

(173-174) “The number of new cases of leprosy among female decreased from 103 in 2011 to 41 in 2020, with the proportion of female leprosy cases fluctuating between 29.95% and 38.51%”

(278-280) “According to spatial distribution analysis, from 2011-2013, the distribution pattern of the NCDR of leprosy showed significant spatial heterogeneity. In 2014, the high NCDR regions were mainly located in southeast, north, southwest regions.”
 
Consideration 1: The results found need better interpretation and discussion. The epidemiological-evolutionary course of leprosy is peculiar and does not change abruptly from one year to another, as shown in this manuscript. There are significant variations in the NCDR from one region to another year to year that may significantly represent a lack of effective surveillance in low-endemic areas and the occurrence of pseudo-silent areas. In the period between 2011 to 2020 there are areas with NCDR/100,000 inhabitants with averages between 2.19 and 4.54, becoming areas with NCDR/100,000 with averages between 0.00 and 0.25, and vice versa. (2).

Thanks for reviewer’s comments.
Revised in discussion line 291-293: In this study, there are significant variations in the NCDR from one region to another year to year that may significantly represent a lack of effective surveillance in low-endemic areas and the occurrence of pseudo-silent areas.(2)

Figure 1 Spatial distribution of newly detected leprosy patients at prefecture and county level in Yunnan ,China 2011-2020.

Consideration 2: Grade 2 disability in the diagnosis of leprosy is another very sensitive epidemiological indicator. It amounted to around 10% between the years 2011 to 2020 in this region, demonstrating that patients remain with advanced disease at the time of diagnosis, perpetuating the disease transmission chain. (3)

Consideration 3: The woman leprosy diagnosis' in this region follows the same trend observed globally, however, the rates fluctuation' in the period was considerable, suggesting underdiagnoses. (4)

Consideration 3: Children with leprosy directly reflect the epidemiological scenario of the disease in a particular region. The large fluctuation in the period evaluated should be widely discussed, also suggesting underdiagnoses. (5).
Reply: Thanks for reviewer’s comments.

Revised in discussion line 281-284: While the female and children proportion with leprosy disease were fluctuating in the study period, suggesting under diagnoses. (4)(5) In addition, The proportion of physical disability in leprosy patients was above 10%, which was very high and demonstrated that patients remain with advanced disease at the time of diagnosis, perpetuating the disease transmission chain. (3)

The number of diagnosed children does not follow the proportion of new multibacillary cases diagnosed, according table one. What do the authors think about this?
Reply: Thanks for reviewer’s comments.

Which may due to the disease characteristics changed from high endemic status to low endemic status.

(280-281): “After 2015, besides the above areas, northwest region became the new high NCDR region”.

Consideration 3: I would like to understand this result highlighted in the illustration provided in atached document.

Reply: Thanks for reviewer’s comments.

Dêqên Tibetan Autonomous Prefecture, a backwater region, located in northwest region of Yunnan China. After 2015, with the intensification of the work, the intensity of case finding in Tibetan areas has increased.

Consideration 4: In regions with epidemiological clusters for leprosy, there is always an expectation of the new cases numbers diagnosed in a given year to the number of new cases that will be detection’ in the following year. These abrupt changes in the epidemiological scenario of leprosy may reinforce a problem of local underdiagnoses and, in part, justify the results found by the authors in this manuscript. (6)

Reply: Thanks for reviewer’s comments.

Revised in discussion line 294-296: These abrupt changes in the epidemiological scenario of leprosy may reinforce a problem of local underdiagnoses and, in part, justify the results found in this study. (6)

Consideration 5: What does the number of “expected cases” expressed in table 5 mean?

According to SaTScan ^TM^ User Guide for version 9.0, with the discrete Poisson model 1 , the number of cases in each location is Poisson-distributed. Under the null hypothesis, and when there are no covariates, **the expected number of cases** in each area is proportional to its population size, or to the person-years in that area. Poisson data can be analyzed with the purely temporal, the purely spatial, the space-time and the spatial variation in temporal trends scan statistics.

Final considerations:

The results of this manuscript are relevant and contribute to the proposed objective. The discussion needs to consider other aspects that promote improvement in local leprosy control actions.
In this way, other locations where leprosy remains a public health problem can benefit these results. The number of leprosy newly cases remains practically stable over the last 12 years.
Obviously, the statistics for the years 2020 and 2021 will not reflect reality when available, taking into account the impact of the pandemic by COVID 19 and the lack of multidrug therapy that occurred during this period. 
This reviewer ends with a question, title of a manuscript referenced below: Global elimination of leprosy by 2020: are we on track? (7)
___________________________________________________________________

References:
1. WHO Technical Report Series No. 874. Geneva: WHO; 1998 (http://www.who.int/iris/handle/10665/42060).
2. Silva CLM, Fonseca SC, Kawa H, Palmer DOQ. Spatial distribution of leprosy in Brazil: a literature review. Rev Soc Bras Med Trop. 2017;50:439-449.
3. Raposo MT, Reis MC, Caminha AVQ, et al. Grade 2 disabilities in leprosy patients from Brazil: Need for follow-up after completion of multidrug therapy. PLoS Negl Trop Dis. 2018;12:e0006645.
4. Price VG. Factors preventing early case detection for women affected by leprosy: a review of the literature. Glob Health Action. 2017;10(sup2):1360550.
5. Oliveira MB, Diniz LM. Leprosy among children under 15 years of age: literature review. An Bras Dermatol. 2016;91:196-203.
6. Gupte MD, Murthy BN, Mahmood K, Meeralakshmi S, Nagaraju B, Prabhakaran R. Application of lot quality assurance sampling for leprosy elimination monitoring--examination of some critical factors. Int J Epidemiol. 2004;33:344-348.
7. Blok DJ, De Vlas SJ, Richardus JH. Global elimination of leprosy by 2020: are we on track? Parasit Vectors. 2015;8:548.

Reviewer #2: The study is very interesting, and will be benefit to the policy made of leprosy control in Yunnan province.

Reviewer #3: Dear Authors,
I read your paper with interest and attention. Knowledge about leprosy clustering is realy important in the manegement of resources and the adoption of adequate strategies for its control. I would like to point some questions to you:
In line 94 Yunnan is referred as a city, please verify
In your analysis and discussion you used the WHO classification, so I suggest to supress the information about RJ classification, mentioned in lines 104 and 105
Reply: Thanks for reviewer’s comments.

“R-J classification” was deleted in line 104-105.

In your results it could be interesting to inform how many children were diagnosed with G2D, if you have any in this period. It could be important to discuss this considering the new goal recomended by WHO. (lines 163- 169)
Reply: Thanks for reviewer’s comments.

According to Global Leprosy Strategy 2016–2020 Monitoring and Evaluation Guide

Accelerating towards a leprosy-free world, the targets are: (1) zero disability among new child cases; (2) reduction of G2D among new cases to less than 1 per million population; and (3) zero countries with legislation allowing discrimination on the basis of leprosy.

For Yunnan, with the highest burden of leprosy in China, there was still G2D in Children in 2018. which indicated that there was still long way to go for elimination burden of leprosy disease.

Table 1. The children of leprosy cases in Yunnan, China, 2011-2020.

| Physical disability | N | % | Year |
| --- | --- | --- | --- |
| Total | 54 | 100.00% | 2011-2020 |
| G0D | 50 | 92.59% | 2011-2020 |
| G1D | 1 | 1.85% | 2011 |
| G2D | 1 | 1.85% | 2018 |
| Missing data | 1 | 1.85% | 2011 |

In line 313 southeast is repeated

Reply: Thanks for reviewer’s comments.

Repeated “southeast” was deleted in line 313.

In my opinion a brief discussion on G2D is lacking. The authors reported a decline in the number of NCDR with G2D, but this is still high. This finding is more robust than the ratio of MB case in the context of late diagnosis. I would like to hear from you about the rise of the ratio of MB cases in the context of progressive and rapid reducing of the disease in this province. In classical studies developed in Norway this finding was reported as a possible epidemiologial indicator of leprosy decline. So when we have a rising in the ratio of MB cases and a pronunced decline in NCDR with G2D maybe we are faced with a situation signaling to decline and not only with the situation of late diagnosis.
Reply: Thanks for reviewer’s comments.

The remarkable progress has been made in the elimination of leprosy in this area. And the rate of physical disability per million population was decreased dramatically. However, the proportion of physical disability was still high in the study region. The rate of early diagnosis was ranged from 53.9 to 60.3% in 1990-2019[ref]. delayed diagnosis still existing.

--------------------

PLOS authors have the option to publish the peer review history of their article (<a href="https://journals.plos.org/plosntds/s/editorial-and-peer-review-process#loc-peer-review-history" target="_blank">what does this mean?</a>). If published, this will include your full peer review and any attached files.<br><br>

If you choose “no”, your identity will remain anonymous but your review may still be made public.<br><br>

<b>Do you want your identity to be public for this peer review?</b> For information about this choice, including consent withdrawal, please see our <a href="https://www.plos.org/privacy-policy" target="_blank">Privacy Policy</a>.

Reviewer #1: No

Reviewer #2: No

Reviewer #3: No


Figure Files:
While revising your submission, please upload your figure files to the Preflight Analysis and Conversion Engine (PACE) digital diagnostic tool, https://pacev2.apexcovantage.com. PACE helps ensure that figures meet PLOS requirements. To use PACE, you must first register as a user. Then, login and navigate to the UPLOAD tab, where you will find detailed instructions on how to use the tool. If you encounter any issues or have any questions when using PACE, please email us at [figures@plos.org](mailto:figures@plos.org).
 
Data Requirements:
Please note that, as a condition of publication, PLOS' data policy requires that you make available all data used to draw the conclusions outlined in your manuscript. Data must be deposited in an appropriate repository, included within the body of the manuscript, or uploaded as supporting information. This includes all numerical values that were used to generate graphs, histograms etc.. For an example see here: http://www.plosbiology.org/article/info%3Adoi%2F10.1371%2Fjournal.pbio.1001908#s5.
 
Reproducibility:
To enhance the reproducibility of your results, we recommend that you deposit your laboratory protocols in protocols.io, where a protocol can be assigned its own identifier (DOI) such that it can be cited independently in the future. Additionally, PLOS ONE offers an option to publish peer-reviewed clinical study protocols. Read more information on sharing protocols at https://plos.org/protocols?utm_medium=editorial-email&utm_source=authorletters&utm_campaign=protocols

References
Please review your reference list to ensure that it is complete and correct. If you have cited papers that have been retracted, please include the rationale for doing so in the manuscript text, or remove these references and replace them with relevant current references. Any changes to the reference list should be mentioned in the rebuttal letter that accompanies your revised manuscript. If you need to cite a retracted article, indicate the article's retracted status in the References list and also include a citation and full reference for the retraction notice.

Those has been noticed.
__________________________________________________
In compliance with data protection regulations, you may request that we remove your personal registration details at any time.  (Use the following URL: https://www.editorialmanager.com/pntd/login.asp?a=r). Please contact the publication office if you have any questions.
